# Living arrangements and life satisfaction: mediation by social support and meaning in life

**DOI:** 10.1186/s12877-020-01541-8

**Published:** 2020-04-15

**Authors:** Yan Lin, Huimin Xiao, Xiuyan Lan, Shuangshuang Wen, Shaoshao Bao

**Affiliations:** 1 School of Nursing, Fujian Medical University, No. 1 Xuefu North Road, University Town, Fuzhou, 3500108 Fujian China; 2 Fujian Provincial Hospital, Fuzhou, China; 3 Fujian Medical University Affiliated Clinical Medical Institute, Fuzhou, China; 4 The Eighth Affiliated Hospital, Sun Yat-sen University, Shenzhen, China

**Keywords:** Living arrangements, Life satisfaction, Social support, Meaning in life, Mediation effect

## Abstract

**Background:**

Living arrangements have impact on life satisfaction among older adults. However, the mechanism how it works has received less attention. This study aims to examine the mediating role of meaning in life and social support in the relationship between living arrangements and life satisfaction.

**M**ethods**:**

A total of 215 older adults from nine nursing homes and three communities were included in this study. The Social Support Rating Scale, Meaning in Life Questionnaire and Life Satisfaction Index A were adopted. Data were analyzed with Hayes’ s bias-corrected bootstrapping method.

**Results:**

Both social support and presence of meaning in life had positive correlations with life satisfaction (*p*<0.001), and they were significant mediators between living arrangements and life satisfaction (*p*<0.01).

**Conclusion:**

To improve the life satisfaction of nursing home residents, more emphasis should be placed on encouraging residents to seek or maintain a meaningful life and creating a more positive climate of social support.

## Background

The global population is aging rapidly. The number of older adults aged 60 years or more is projected to reach two billion by 2050, or 22% of the world population [1]. China has the largest elderly population in the world. In Fuzhou of China, the number of people aged 60 years or above is estimated at 1.51 million, comprising 17% of the total population [2]. Older adults often experience reductions in physical function, cognition, personal autonomy and engagement in social activities [3, 4]. In many cases, the decision by older adults to relocate to a nursing home, made in consultation with their family, is inevitable. Compared to previous generations, modern Chinese nuclear families are less able to care for aging parents. Older adults in China, therefore, are increasingly moving to nursing homes, despite the fact that the vast majority would prefer to remain in their own home [5]. Regardless of where older adults live, life satisfaction is regarded as a key indicator of successful aging [6]. There is a growing interest in exploring how living arrangements affect older adults’ life satisfaction, which may contribute to developing and applying strategies for successful aging.

Life satisfaction is defined as an individual’s evaluation of his or her attitudes and feelings about life, based on a standard that he or she has set [7]. Convoy model of social relations [8] and Novena’s conceptual model of meaning in life [9] further reveal the relationships of life satisfaction, living arrangements, social support and meaning in life. The convoy model indicates that social support from different social networks (e.g. living arrangements) plays an important role in determining individual subjective well-being [8], such as life satisfaction. According to the latter model, the conditions (e.g. living arrangements) can serve as a foundation for the components of meaning in life. Feeling happy, satisfied and joyful should be the outcomes of having meaning in life [9]. Theoretically, social support and meaning in life could be the mediators of living arrangements of life satisfaction.

Numerous studies have identified the predictors of life satisfaction among older adults [10–12]. Living arrangements seem to be a fundamental factor related to life satisfaction [13]. A previous study has indicated that community-dwelling older adults showed higher levels of life satisfaction than nursing home residents [14]. This is further confirmed by another study that showed the majority of older adults living in nursing homes had low levels of life satisfaction [15]. Social support could be a significant factor for life satisfaction in older adults too. There is evidence that social support is consistently found to be positively correlated with life satisfaction in older adults [16, 17]. It has emerged as a crucial protective factor for life satisfaction among them [18, 19]. Meaning in life has been also revealed as another significant factor. As motivation and goals can predict positive expectancies, meaning in life may predict enhanced life satisfaction. It has been demonstrated that the presence of a higher level of meaning in life contributes to increasing levels of life satisfaction [20]. Another study also has found that when a sense of meaning in life decreases, satisfaction with life tends to be lower [21].

In addition, social support and meaning in life are also associated with a person’s living arrangements. Numerous studies have illustrated significant differences in the social support that is available to people depending on their living arrangements. For example, Su et al. [22] report that empty nesters living in rural areas have access to higher levels of social support than those living in urban areas. Muramatsu, Yin and Hedeker [23] have also revealed that social support is more available to older adults living in a state that is supportive of home- and community-based services. Furthermore, nursing home residents may enjoy less social support, compared to those living with their families. This is mainly due to the fact that moving to a nursing home may lead to the loss of intimate attachments [24]. Among older adults, living arrangements also play an important role in meaning in life. Previous studies have found that older adults who are institutionalized have a lower sense of meaning in life than community-dwelling older adults [25, 26].

**Fig. 1:**
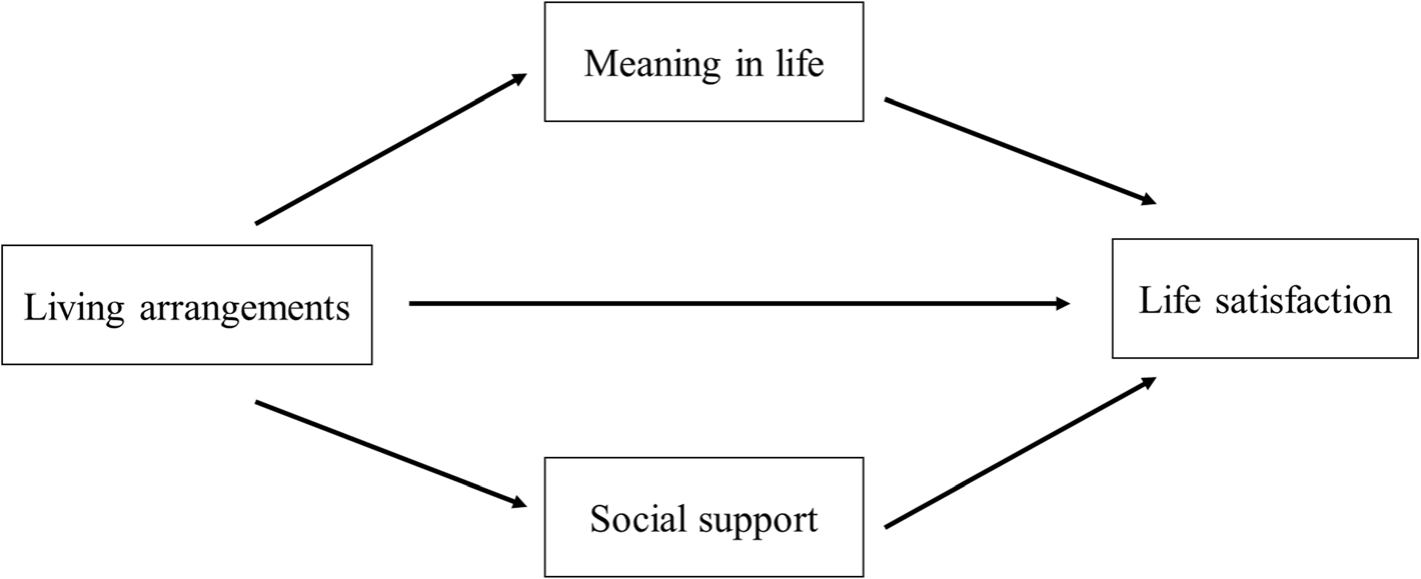
Conceptual model proposed for this study

Although many studies have found a significant relationship among living arrangements, social support, meaning in life and life satisfaction in older adults, to date, no study has examined the effect of social support and meaning in life on the relationship between living arrangements and life satisfaction in older adults. Based on the above mentioned theoretical and empirical background, this study proposes a conceptual model (Fig. 1) to link these variables with life satisfaction. It is hypothesized that living arrangements exerted a direct effect on life satisfaction and an indirect effect mediated by social support and meaning in life. This study aims to provide a new insight into the mediating role of meaning in life and social support between living arrangements and life satisfaction among older adults.

## Methods

### Study design and participants

This was a cross-sectional survey. A total of 215 older adults were conveniently recruited between May 2016 and April 2017. The inclusive criteria were: (a) nursing home residents who had been living in a nursing home for more than 6 months or community-dwelling older adults who were permanent local residents; (b) aged 60 years or older; (c) ability to communicate. The exclusion criteria were as follows: (a) older adults with cognitive impairment, as measured by the Short Portable Mental Status Questionnaire (cut off ≥2) [27], and (b) critically ill or in the terminal stage of illness.

### Variables and measures

#### Demographic variables

The sociodemographic variables included age, gender, education level, marital status, income source, occupation, activities of daily living, chronic disease and religion.

#### Independent variable

Living arrangement (community vs. nursing home) was the independent variable in this study. The community-dwelling older adults in this study usually lived in their own home and received necessary medical care at a community health center [28]. The nursing home residents received institutional care, including personal care, recreational activities and medical care.

#### Mediating variables

Meaning in life and social support were two mediating variables in this study. Meaning in life was measured by Meaning in Life Questionnaire (MLQ), consisting of two five-item subscales, Presence (MLQ-P; perceived meaning) and Search (MLQ-S; motivation to discover meaning) [29]. The Presence subscale measures an individual’s perception of the degree to which his or her life is meaningful, such as “I understand my life’s meaning”. The Search subscale measures an individual’s motivation to find meaning in life, such as “I am searching for meaning in my life.” All items are rated on a seven-point scale ranging from 1 (absolutely untrue) to 7 (absolutely true). Scores of each subscale range from 5 to 35. The higher scores indicate higher levels of presence of meaning or search for meaning. The Chinese translation of MLQ was used in this study. Its reliability was confirmed with alpha coefficient estimates of internal consistency of 0.81 and 0.84, respectively [30]. Social support was assessed using the Social Support Rating Scale [31], which has widely been used among different populations in China [32, 33]. Social support was examined from three dimensions with 10 items: objective support, subjective support, and utilization of support. The scores for the scale range from 12 to 66. A higher score predicts more social support. The Cronbach’s α for the scale was 0.83 [34].

#### Dependent variable

In this study, life satisfaction was measured using the Life Satisfaction Index A [35]. The index consists of 20 “agree” or “disagree” attitude items. It assigns one point for positive answers, and no points for negative answers or “don’t know” answers. Each item scores 0 to 1 and provides a range of 0 to 20 in total. Higher total scores indicate higher levels of satisfaction. The Cronbach’s α for the Chinese version scale was 0.74 [36, 37].

### Data collection

The ethic approval of this study was obtained from the co-responding author’s university. Then, our study proposal was sent to the available community committees and nursing homes in Fuzhou to invite them to participate in this study. Nine nursing homes and three communities agreed to be recruited for this study. After the informed consent was obtained, the individual face-to-face interviews were conducted by senior undergraduate nursing students with experience in conducting surveys. The data collectors read each question aloud and objectively marked the participants’ answers in the questionnaire. Each interview last about 20 min .

### Data analysis

Descriptive statistics were calculated for all of the targeted variables. Categorical variables were described by frequencies and percentage, and continuous variables, by mean and standard deviation. A chi-square test was used to examine the differences in demographic characteristics between community-dwelling older adults and nursing home residents. An independent-sample t-test was used to compare life satisfaction, presence of meaning, search for meaning and social support between the two groups. Additionally, correlational analyses were calculated for life satisfaction, presence of meaning, search for meaning, and social support.

Hayes’s bias-corrected bootstrapping method [38] was employed to examine the multiple mediation effects of presence of meaning, search for meaning, and social support. The reason is that although a causal steps approach and the Sobel test enjoy some application in test mediation effects, they have a major flaw [39, 40]. They require the assumption that the sampling distribution of the indirect effect is normal. But the sampling distribution of indirect effect tends to be asymmetric, with nonzero skewness and kurtosis [41]. Indeed, bootstrapping has more power and better Type I error control than the Sobel test and the causal steps approach [38]. We used 5000 bootstrap samples, and bias was corrected at 95% confidence intervals (CI) to calculate the indirect effect of each variable. If the CI of the indirect effect did not include zero, it indicated that the indirect effect was significant.

## Results

### Demographic characteristics of study participants

Table 1 presented the sample characteristics by comparing the frequencies or means between the two settings. There were 101 respondents from long-term nursing homes, and 114 from communities. The mean age of all participants was 75.81 (SD = 9.42). The proportion of nursing home residents aged 75 or above was significantly larger than that of community residents (*p*<0.001). Nearly half of the respondents were married (45.6%), however, the proportion of married elderly living in the community (64.9%) was significantly larger than that of those living in nursing homes (23.8%). A total of 71.2% of respondents were living on a pension, while the rest were living on savings, public assistance or financial support from their children. There was a slightly lower proportion of nursing home residents living on a pension, compared with their counterparts (61.4% vs 79.8%). A χ^2^ test indicated that no significant differences between the two groups were found in terms of gender, occupation, education level, religion or chronic disease.

**Table 1:** Participant demographic characteristics

Variable	Total(*n* = 215) n(%)	Nursing Home(*n* = 101) n(%)	Community(*n* = 114) n(%)	*X*^2^	p
Age (years)				−7.076^***^	0.000
60–74	102 (47.4)	22 (21.8)	80 (70.2)		
≥75	113 (52.6)	79 (78.2)	34 (29.8)		
Marital status				−6.032^***^	0.000
Married	98 (45.6)	24 (23.8)	74 (64.9)		
Other marital status	117 (54.4)	77 (76.2)	40 (35.1)		
Source of income				−2.972^**^	0.003
Pension	153 (71.2)	62 (61.4)	91 (79.8)		
Other	62 (28.8)	39 (38.6)	23 (20.2)		
Gender				−0.353	0.724
Male	90 (41.9)	41 (40.6)	49 (43)		
Female	125 (58.1)	60 (59.4)	65 (57)		
Occupation				−0.954	0.340
Civil servant	39 (18.1)	19 (18.8)	20 (17.5)		
Employee	91 (42.3)	37 (36.6)	54 (47.4)		
Other	85 (39.5)	45 (44.6)	40 (35.1)		
Education level				−0.678	0.498
Primary school	105 (48.8)	52 (51.5)	53 (46.5)		
Middle school	54 (25.1)	24 (23.8)	30 (26.3)		
High school or above	56 (26.1)	25 (24.8)	31 (27.2)		
Chronic disease				−1.526	0.127
No	84 (39.1)	34 (33.7)	50 (43.9)		
Yes	131 (61.9)	67 (66.3)	64 (56.1)		
Religion				−0.746	0.456
No	108 (50.2)	48 (47.5)	60 (52.6)		
Yes	107 (49.8)	53 (52.5)	54 (47.4)		

^**^p<0.01(two-tailed); ^***^p<0.001(two-tailed)

### Descriptive statistics and correlations among the main variables

Table 2 showed that social support, presence of meaning, search for meaning, and life satisfaction was different between nursing home residents and community-dwelling older adults(*p*<0.001). The nursing home residents reported lower levels of social support, presence of meaning, search for meaning and life satisfaction, compared with the community-dwelling older adults (*p*<0.001). All three variables were significantly correlated with one another. Social support was positively related to presence of meaning (r = 0.261, *p*<0.001), search for meaning (r = 0.160, *p*<0.05) and life satisfaction (r = 0.359, *p*<0.001). Higher level of presence of meaning and search for meaning were all significantly associated with a higher level of life satisfaction (r = 0.450, *p*<0.001; r = 0.397, *p*<0.001).

**Table 2:** Descriptive statistics and correlations among variables (n = 215)

Variable	Nursing home (n = 101)		Community (n = 114)		t	Correlations among variables			
	M	SD	M	SD		1	2	3	4
(1) Social support	28.04	6.84	35.17	7.58	−7.21^***^	1.000	.261^**^	.160^*^	.359^**^
(2) Presence of meaning	17.41	7.79	21.79	8.76	−3.85^***^	–	1.000	.745^**^	.450^**^
(3) Search for meaning	15.98	8.76	19.10	6.53	−3.60^***^	–	–	1.000	.397^**^
(4) Life satisfaction	11.19	3.97	13.65	3.77	−4.66^***^	–	–	–	1.000

*M* mean, *SD* standard deviation; ^**^p<0.01(two-tailed); ^***^p<0.001(two-tailed)

### Mediating effects of social support, presence of meaning, and search for meaning

**Fig. 2:**
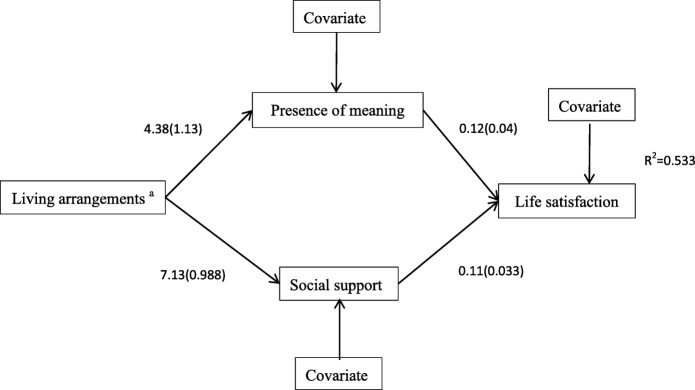
Results of final path model (N = 215). Note. Higher scores of life satisfaction, social support and presence of meaning scores indicate better status; Covariates for presence of meaning (age, martial status, source of income, gender,occupation, education, chronic disease status, and religion), social support (age, martial status, source of income, gender,occupation, education, chronic disease status, and religion), and life satisfaction (age, martial status, source of income, gender,occupation, education, chronic disease status, and religion) were included in the path model. a. The reference group was a nursing home setting

The findings about the mediation effects of presence of meaning, search for meaning, and social support on the relationship between living arrangements (nursing home vs. community) and life satisfaction were presented in Table 3, and the final path model was presented in Fig. 2. The total effect of living arrangements on life satisfaction was significant (b = 2.460, SE = 0.528, *p*<0.001, bias-corrected and percentile 95% CI = [1.419, 3.501]). The direct effect of living arrangements on life satisfaction was statistically non-significant (*p*>0.05, bias-corrected and percentile 95% CI = [− 0.210,1.899]). Both presence of meaning and social support were significant in mediating the relationship between living arrangements and life satisfaction (b = 0.531, SE = 0.219, *p*<0.05, bias-corrected 95% CI = [0.182, 1.054], percentile 95% CI = [0.163, 1.010]; b = 0.794, SE = 0.274, *p*<0.01, bias-corrected 95% CI = [0.306, 1.398], percentile 95% CI = [0.311, 1.290], respectively). Recording to the result of percentile, that showed the statistical analysis of interval contained 0, search for meaning was not significant in mediating the relationship between living arrangements and life satisfaction(b = 0.281, SE = 0.166, *p*<0.05, bias-corrected 95% CI = [0.027, 0.694], percentile 95% CI = [− 0.008, 0.647]).

**Table 3:** Mediation effects of social support and life meaning on relationship between living arrangements and life satisfaction (n = 215)

				Bootstrapping			
	Point Estimate	Product of coefficients		Bias Corrected 95% CI		Percentile 95% CI	
		SE	Z	Lower	Upper	Lower	Upper
Total effects							
Living arrangement → Life satisfaction	2.460	0.528	4.659^***^	1.419	3.501	1.419	3.501
Direct effects							
Living arrangement → Life satisfaction	0.845	0.535	1.579	−0.210	1.899	−0.210	1.899
Indirect effects							
Total Indirect Effects	1.615	0.364	4.437^***^	0.994	2.448	0.945	2.386
Living arrangement → Presence of meaning → Life satisfaction	0.531	0.219	2.309^*^	0.182	1.054	0.163	1.010
Living arrangement → Search for meaning → Life satisfaction	0.281	0.166	1.624	0.027	0.694	−0.008	0.647
Living arrangement → Social support→ Life satisfaction	0.794	0.274	2.819^**^	0.306	1.398	0.311	1.290

^*^p<0.05(two-tailed);^**^p<0.01(two-tailed); ^***^p<0.001(two-tailed)

## Discussion

This was a descriptive study to explore how living arrangements affect life satisfaction in older adults. It confirmed that community-dwelling older adults had a higher level of life satisfaction than nursing home residents. Importantly, it further revealed that the effect of living arrangements on life satisfaction is totally mediated by presence of meaning in life and social support.

The t-test in this study showed that community-dwelling older adults reported higher levels of life satisfaction, higher levels of social support and greater meaning in life than those living in a nursing home. The more comprehensive life satisfaction path model further indicated that their living arrangements did not directly influence life satisfaction, but had a totally indirect influence through different social and spiritual status. This finding suggested that living in a nursing home did not cause poor life satisfaction, but that mediating factors (social support and presence of meaning in life) exerted an important impact on life satisfaction. The possible reason for community-dwelling older adults with a higher life satisfaction may be also due to place attachments. Scannell and Gifford have revealed that places can emerge various individual and cultural meaning from personally important experiences [42]. This point is consistent with aging in place, which indicates the importance of creating sustainable environments [43].

### The mediating effects of presence of meaning

The present study indicated that the relationship between living arrangements and life satisfaction was mediated by presence of meaning in life. Specifically, the community-dwelling older adults reported higher levels of meaning in life, which was associated with better life satisfaction, compared with nursing home residents. This is supported by Glaw et al., who identified a positive association between meaning in life and life satisfaction [20]. Presence of meaning in life refers to the extent to which people comprehend, make sense of, or perceive significance in their lives, as well as the degree to which they view themselves as having a purpose or mission [29]. It may be that presence of meaning in life was related to higher levels of well-being and mental health throughout the human life span [44]. It may also be possible that presence of meaning in life can protect individuals from negative outcomes [45], and make them less vulnerable to psychopathology [46]. Another explanation could be that individuals with higher levels of meaning in life can cope more effectively with physical health challenges and overcome obstacles more easily [47].

### The mediating effects of social support

This study further revealed that a positive relationship between living arrangements and life satisfaction was mediated by social support. It suggested that compared to nursing home residents, community-dwelling older adults enjoy higher levels of social support, which was associated with better life satisfaction. Cumulative evidence showed that social support contributed to improved psychological well-being [48]. In this study, it may be that community-dwelling older adults receive more support than older adults living in nursing homes, particularly physical, psychological, emotional, and financial support from their family and children [49–51]. Being deeply engaged with family members contributes to higher levels of life satisfaction. It may also be possible that social support can alleviate the negative effects of stressful events, contributing to greater life satisfaction [52]. Older adults face negative events, such as a reduction in physical functioning and cognition, an increase in dependency and the death of loved ones. Social support was found to be a protector from the negative effects of age-related challenges in older adults [53, 54]. Thus, older adults who are more engaged in social support networks are more likely to be satisfied with their lives.

These study findings are particularly important, which open a new nursing perspective, because the mediating effects on the relationship between living arrangements and life satisfaction provide evidence for improving nursing home residents’ life satisfaction. Given the importance of meaning in life, logotherapy could be a likely contributor to stimulating nursing home residents’ will to find meaning in life through three different ways including“creative values,” “experiential values,” and “attitudinal values” [55, 56]. Nursing staff could also guide older adults to set small goals, and help them fulfill these goals in their daily lives. They could facilitate residents to offer reminiscence activities and contributions, and help them clarify their values and what brings them meaning in life. Maximizing the social integration of the residents are also necessary. Nursing home staff could hold a variety of activities to increase residents’ opportunities to interact with one another and encourage them to engage in more social activities. In particular, for those who are less mobile, nursing home staff could offer opportunities for interaction by escorting them to other residents’ rooms, and maximize the opportunities for meeting other residents during activities, not by solely sitting them next to each other. Ensuring good relationships between nursing home staff and residents would be another effective approach to improve social support for nursing home residents. Maintaining close ties with family and friends, are also good options for enhancing their life satisfaction. Following this, researchers might explore how older adults place attachment-related resilience and/or belonging in relation to life satisfaction.

### Limitations

The results and implications of this study should be considered in light of several limitations. First, the data were collected at one point in time, and this study did not examine the modeled relationships longitudinally or test the models’ predictive power over time. Second, this study only 215 older adults from nine nursing homes and three communities in southeastern China. The findings’ generalizability is limited. Finally, the sole use of self-reported measures and a convenience sampling procedure are also methodological limitations, it could have been strengthened to use a mixed methods involving interviews with participants would provide more context to complement the quantitative findings.

## Conclusion

The study provides additional evidence of the importance of presence of meaning in life and social support for life satisfaction in older adults from different living arrangements. To improve the life satisfaction of nursing home residents, more emphasis should be placed on encouraging residents to seek or maintain a meaningful life and on creating a more positive climate of social support.

## Data Availability

The data that support the findings of this study are available from the corresponding author on request.

## Abbreviations

MLQ: Meaning in life questionnaire
MLQ-P: Meaning in life questionnaire-presence
MLQ-S: Meaning in life questionnaire-search
CI: Confidence interval
M: Mean
SD: Standard deviation
SE: Standard Error
